# Efficacy and safety of Xuesaitong soft capsule plus antiplatelet agents in ischemic stroke patients: a large-scale, multicenter, controlled, retrospective real-world study

**DOI:** 10.3389/fphar.2026.1851121

**Published:** 2026-09-03

**Authors:** Haiqing Song, Yanjie Gao, Yuan Tang, Xinghui Gao, Longfei Wu, Li Li, Quanxi Ge, Zhigang Chen, Ting He

**Affiliations:** 1 Department of Neurology, Xuanwu Hospital, Capital Medical University, Beijing, China; 2 Department of Neurology, Dongfang Hospital, Beijing University of Chinese Medicine, Beijing, China; 3 Department of Rehabilitation Medicine, Dongfang Hospital, Beijing University of Chinese Medicine, Beijing, China; 4 Acupuncture Research Institute, First Teaching Hospital of Tianjin University of Traditional Chinese Medicine, Tianjin, China; 5 National Clinical Research Center for Traditional Chinese Medicine Acupuncture and Moxibustion, Tianjin, China; 6 Department of Pharmacy, Dongfang Hospital, Beijing University of Chinese Medicine, Beijing, China

**Keywords:** antiplatelet agents, efficacy, ischemic stroke, safety, Xuesaitong soft capsule

## Abstract

**Background:**

Previous experimental studies have suggested synergistic effects of *Panax notoginseng* saponins combined with antiplatelet agents in ischemic stroke. However, large-scale real-world clinical evidence regarding the efficacy and safety of this combination remains limited. This study aimed to explore the efficacy and safety of Xuesaitong soft capsule plus antiplatelet agents in ischemic stroke patients.

**Methods:**

This multicenter, controlled, retrospective real-world study included 38,652 patients with ischemic stroke who received either antiplatelet agents (aspirin and/or clopidogrel) alone (control group, N = 37,249) or Xuesaitong soft capsule plus antiplatelet agents (observational group, N = 1,403). Propensity score matching (PSM) was initially performed at a 1:20 ratio (N = 1,401 in the observational group vs. N = 27,955 in the control group). A 1:4 PSM sensitivity analysis was subsequently performed to evaluate the robustness of the findings (N = 1,401 in the observational group vs. N = 5,604 in the control group).

**Results:**

After 1:20 matching, the 1-year major adverse cardiovascular and cerebrovascular event (MACCE) incidence was lower in the observational group than in the control group (14.99% vs. 18.71%) (*P* < 0.001). The 2- and 3-year MACCE incidences showed the same trend (both *P* < 0.001). Subgroup analyses indicated that the reduction in 1-year MACCE incidence was greater in patients aged 45–64 years, treated within 2 weeks after stroke onset, with MACCE occurring within 1 month after treatment, and without hypertension, hyperlipidemia, or coronary heart disease (all *P* < 0.05). The incidence of adverse events of special interest was lower in the observational group than in the control group (18.56% vs. 21.82%) (*P* = 0.004). The 1:4 PSM sensitivity analysis confirmed the robustness of the findings, with lower 1-year MACCE incidence in the observational group than in the control group (14.99% vs. 18.58%) (*P* = 0.002).

**Conclusion:**

Xuesaitong soft capsule combined with antiplatelet agents is associated with lower MACCE incidence and gastrointestinal adverse events compared to antiplatelet agents alone in ischemic stroke patients.

## 1 Introduction

Ischemic stroke, a leading cause of mortality and disability worldwide, contributes to a substantial global health burden (Diseases and Injuries, 2024). The current standard treatments for ischemic stroke include intravenous thrombolysis and mechanical thrombectomy within certain therapeutic windows (≤4.5 h for thrombolysis and ≤24 h for thrombectomy) (Chen et al., 2024; Hilkens et al., 2024; Ho and Powers, 2025; Sharma and Lee, 2025; Tsivgoulis et al., 2023; Widimsky et al., 2023). For ischemic stroke patients who are ineligible for recanalization therapies, antiplatelet agents, such as aspirin and clopidogrel, should be applied (Greco et al., 2023; Ho and Powers, 2025). Nevertheless, even after antiplatelet therapies, some patients still experience major adverse cardiovascular and cerebrovascular events (MACCE) (Vallejo-Vaz et al., 2024). On the other hand, the use of these antiplatelet agents raises safety concerns, such as increased risk of gastrointestinal bleeding (Del Giovane et al., 2021; Hilkens et al., 2021; Virk et al., 2023). Therefore, exploring adjunctive therapies to antiplatelet agents that can enhance efficacy while minimizing safety concerns is fundamental to improving the management of ischemic stroke patients.

*Panax notoginseng* saponins (PNSs), the primary bioactive ingredients of *P. notoginseng*, comprise a group of triterpenoid saponins, with notoginsenoside R1 and ginsenosides Rg1, Rb1, and Rd being the most pharmacologically active components (Li et al., 2025). Several studies demonstrated that PNSs could attenuate the progression of ischemic stroke (Gao J. et al., 2022; Xiao et al., 2024; Xu et al., 2021; Zhang et al., 2024). Importantly, previous studies showed that PNSs plus antiplatelet agents could enhance the efficacy and reduce adverse events in ischemic stroke (Cui et al., 2024; Xu et al., 2021; Zhu et al., 2021). For instance, a previous study indicated that PNSs plus aspirin and clopidogrel reduced middle cerebral artery occlusion (MCAO)/reperfusion injury compared to aspirin plus clopidogrel in rats (Zhu et al., 2021). Another study reported that PNSs plus aspirin reduced adverse gastrointestinal effects in MCAO rats (Xu et al., 2021).

Xuesaitong, a blood-activating and stasis-dispelling medicinal product, is one of the most commonly used PNS preparations for treating ischemic stroke (Li et al., 2022). Previous studies and meta-analyses demonstrated that Xuesaitong soft capsules had satisfactory efficacy and safety for the treatment of ischemic stroke (Gao Y. et al., 2022; Wu et al., 2023). For instance, a randomized controlled trial reported that Xuesaitong soft capsules increased functional independence at 3 months compared to placebo in ischemic stroke patients, with comparable safety profiles (Wu et al., 2023). However, most of the existing evidence derives from randomized controlled trials, which may lack generalizability to real-world conditions due to strict eligibility criteria. On the other hand, the potential of Xuesaitong soft capsule plus antiplatelet agents for the treatment of ischemic stroke remains to be investigated.

Therefore, this large-scale, multicenter, controlled, retrospective real-world study aimed to compare the efficacy and safety of Xuesaitong soft capsule plus antiplatelet agents vs. antiplatelet agents in ischemic stroke patients.

## 2 Materials and methods

### 2.1 Patients

This multicenter, controlled, retrospective real-world study screened 38,652 patients diagnosed with ischemic stroke who received antiplatelet agents or Xuesaitong soft capsule combined with antiplatelet agents between 1 January 2017, and 31 December 2021. All data were retrieved from the Tianjin Health Medical Big Data Super Platform. The inclusion criteria were: i) Age ≥18 years without sex restriction; ii) First diagnosis with ischemic stroke between 1 January 2017, and 31 December 2021; iii) Use of the research drugs for at least 2 months within 1 year after diagnosis or at least 1 month within 6 months. The exclusion criteria were: i) Use of the research drugs before their first diagnosis of ischemic stroke; ii) Comorbid malignant tumors; iii) Use of Xueshuantong capsule, compound Xueshuantong capsule, or Sanqitongshu capsule (a preparation enriched in protopanaxatriol saponins rather than total PNSs) for at least 2 months within 1 year after diagnosis; iv) Missing important information, such as disease diagnosis and treatment information. Furthermore, patients receiving any treatment other than research regimens were excluded: i) Xuesaitong soft capsule only; ii) Naoxintong capsule combined with antiplatelet agents; iii) Tongxinluo capsule combined with antiplatelet agents. This study was approved by the Institutional Review Boards of Xuanwu Hospital, Capital Medical University (Approval No. 2024-099-001) and Dongfang Hospital, Beijing University of Chinese Medicine (Approval No. JDF-IRB-2023040702). The requirement for informed consent was waived by the Institutional Review Boards because this was a retrospective study. This study was conducted in accordance with the principles of the Declaration of Helsinki. All patient data were anonymized prior to analysis, and no personally identifiable information was accessible to the investigators.

### 2.2 Treatment

Based on the treatment regimen, patients were divided into an observational group and a control group. Patients in the control group received antiplatelet agents for antiplatelet therapy (aspirin and/or clopidogrel). Patients in the observational group received Xuesaitong soft capsule combined with antiplatelet agents. Xuesaitong soft capsules (KPC Pharmaceuticals, Inc., Kunming, China) contain PNSs as the sole active pharmaceutical ingredient. The soft capsules were prepared by mixing PNSs with polyethylene glycol 400, glycerol, and purified water. PNSs are extracted from the dried roots and rhizomes of *P. notoginseng* (Burk.) F.H. Chen (Araliaceae), which consist primarily of notoginsenoside R1 and ginsenosides Rg1, Re, Rb1, and Rd. According to the Chinese Pharmacopoeia (2020 edition), Xuesaitong preparations are standardized to contain not less than 5% notoginsenoside R1, 25% ginsenoside Rg1, 2.5% ginsenoside Re, 27% ginsenoside Rb1, and 5% ginsenoside Rd. These five saponins together account for at least 75% of the total content in oral formulations. The identity and quality of the product were verified according to the Chinese Pharmacopoeia using high-performance liquid chromatography (HPLC), with chromatographic identification based on comparison with authenticated reference extract and reference standards. All Xuesaitong soft capsules included in this study were manufactured by KPC Pharmaceuticals, Inc. (Kunming, China). Each production batch underwent HPLC fingerprint-based quality control according to internal quality standards, ensuring high batch-to-batch consistency. These product-specific characteristics were obtained from the official product information, rather than from the original clinical records.

### 2.3 Outcomes

The primary outcome was the 1-year MACCE rate, which was defined as the percentage of patients who experienced a MACCE within 1 year among all eligible patients. MACCE encompassed ischemic stroke, hemorrhagic stroke, transient ischemic attack, myocardial infarction, angina pectoris, acute coronary syndrome, use of thrombolytic drugs, coronary stent placement, intracranial stent placement, and revascularization treatment (thrombectomy, embolus removal, etc.). The secondary outcomes included 2-year MACCE rate, 3-year MACCE rate, 1-year mortality, 1-year visit rate to the rehabilitation department or rehabilitation hospital, accumulating MACCE rate, and adverse events of special interest (AESI).

### 2.4 Statistics

All statistical analyses were conducted using the SAS 9.4 statistical analysis software for programming calculations. Propensity score matching (PSM) was conducted using the nearest neighbor or caliper matching method without replacement, with a ratio of 1:20. The matching covariates included age, gender, type of visit, type of stroke, comorbidity, and concomitant medication. The initial 1:20 PSM strategy was selected because the observational group was substantially smaller than the control group (1,403 vs. 37,249 patients). Given the large available control population, we aimed to maximize the utilization of eligible controls while maintaining adequate covariate balance, thereby improving the precision of treatment effect estimation in this real-world cohort. A sensitivity analysis was performed using an alternative PSM ratio of 1:4 based on the same matching covariates. Multivariable logistic regression analysis on 1-year MACCE risk was conducted to assess whether the between-group difference remained after adjustment for age, gender, type of visit, type of stroke, comorbidity, and concomitant medication. For continuous variables, the number of cases, mean, standard deviation (SD), median, 25th and 75th percentiles, minimum, and maximum were used for description, and the t-test or the Wilcoxon rank sum test was employed for inter-group comparison. For discrete variables, the number of cases and the percentage were used for statistical description. If necessary, a 95% confidence interval (CI) was added, and the χ^2^ test or Fisher’s exact test was adopted for inter-group comparison. Subgroup analyses were predefined according to clinically relevant characteristics, including age, type of stroke, duration from stroke occurrence to treatment, duration from treatment to MACCE, and major comorbidities (hypertension, hyperlipidemia, diabetes, and coronary heart disease). Benjamini-Hochberg False Discovery Rate (BH-FDR) method was used to adjust multiple comparisons. The statistical tests were conducted using two-tailed tests, and a *P* value less than 0.05 was used as the criterion for determining a statistically significant difference between groups.

## 3 Results

### 3.1 Study flow

**FIGURE 1:**
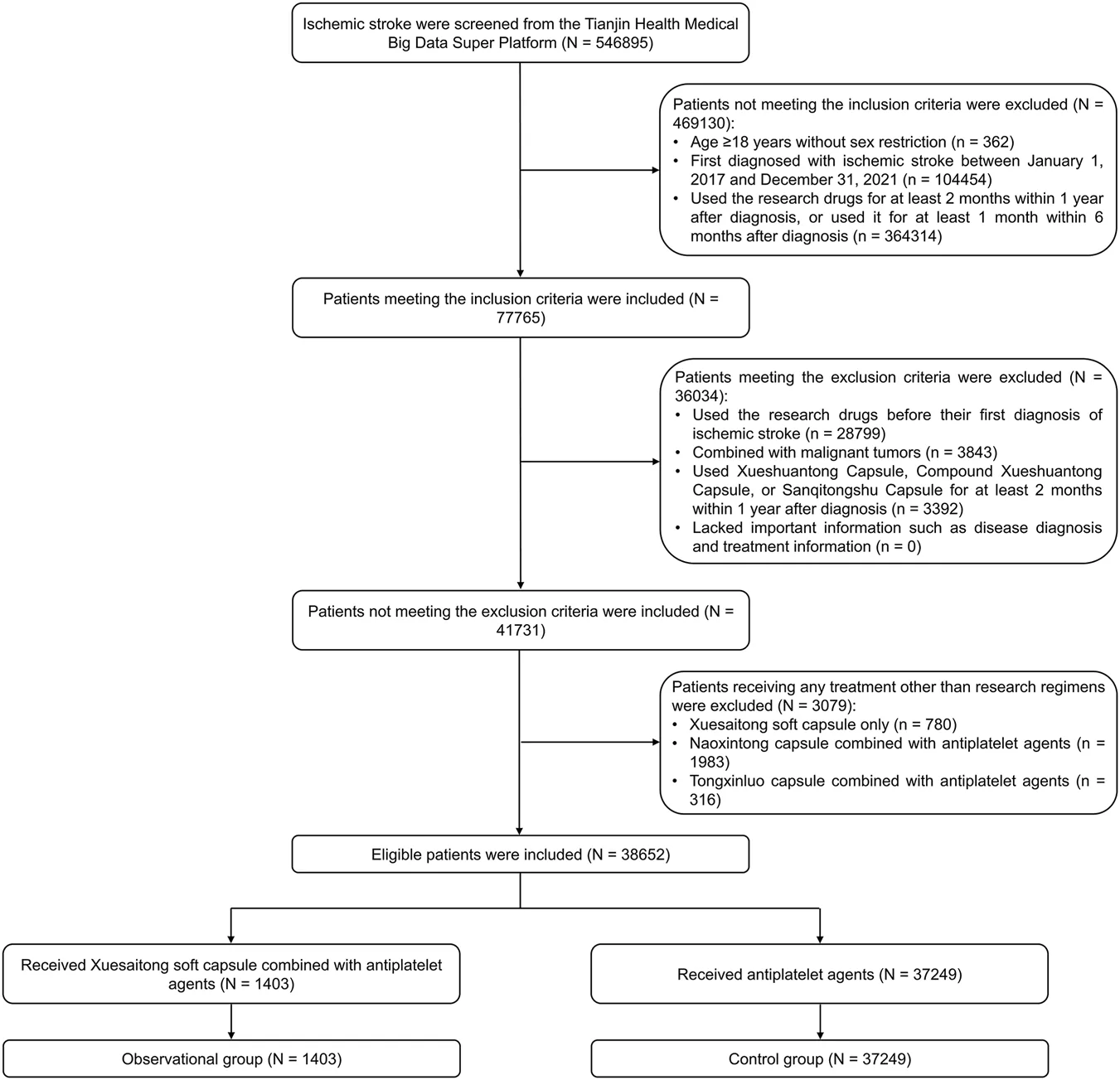
Flowchart.

A total of 546,895 ischemic stroke patients were screened from the Tianjin Health Medical Big Data Super Platform, and 469,130 patients who did not meet the inclusion criteria were excluded. Among the remaining 77,765 patients, 36,034 patients were further excluded for meeting the exclusion criteria. Then, a total of 3,079 patients were excluded for receiving other regimens, including Xuesaitong soft capsule monotherapy, Naoxintong capsule combined with antiplatelet agents, and Tongxinluo capsule combined with antiplatelet agents. Finally, 38,652 patients were enrolled, among whom 1,403 patients receiving Xuesaitong soft capsule combined with antiplatelet agents were assigned to the observational group, and 37,249 patients receiving antiplatelet agents were assigned to the control group (Figure 1).

### 3.2 Clinical features

Before PSM, differences in age (*P* = 0.014), sex (*P* = 0.002), type of medical visit (*P* < 0.001), type of stroke (*P* = 0.007), comorbidities (*P* < 0.001), and concomitant medication (*P* = 0.003) existed between the observational and control groups. After PSM, there were 1,401 patients in the observational group and 27,955 patients in the control group. No differences in age, sex, type of medical visit, type of stroke, and concomitant medication were observed after PSM between the two groups (all *P* > 0.05). However, comorbidities differed between the two groups (*P* < 0.001). The detailed clinical characteristics of the two groups before and after PSM are shown in Table 1.

**TABLE 1:** Clinical characteristics.

Items	Before PSM			After PSM		
	Observational group (N = 1,403)	Control group (N = 37,249)	*P* value	Observational group (N = 1,401)	Control group (N = 27,955)	*P* value
Age (years)						
Patients (missing cases)	1,403 (0)	37,249 (0)	0.014^*^	1,401 (0)	27,955 (0)	0.595^*^
Mean (SD)	62.6 (11.3)	63.4 (11.8)		62.6 (11.3)	62.8 (11.3)	
Median (Q1, Q3)	63.0 (55.0, 70.0)	64.0 (56.0, 71.0)		63.0 (55.0, 70.0)	63.0 (56.0, 70.0)	
Min, max	31, 120	19, 121		31, 120	21, 121	
Sex						
Patients (missing cases)	1,401 (2)	37,208 (41)	0.002^#^	1,401 (0)	27,955 (0)	0.977^#^
Male	916 (65.38)	22,798 (61.27)		916 (65.38)	18,267 (65.34)	
Female	485 (34.62)	14,410 (38.73)		485 (34.62)	9,688 (34.66)	
Type of medical visit						
Patients (missing cases)	1,403 (0)	37,249 (0)	<0.001^#^	1,401 (0)	27,955 (0)	0.719^#^
Emergency	291 (20.74)	6,870 (18.44)		291 (20.77)	5,568 (19.92)	
Outpatient	804 (57.31)	18,105 (48.61)		803 (57.32)	16,125 (57.68)	
Inpatient	308 (21.95)	12,274 (32.95)		307 (21.91)	6,262 (22.40)	
Type of stroke						
Patients (missing cases)	1,403 (0)	37,249 (0)	0.007^#^	1,401 (0)	27,955 (0)	0.146^#^
Non-cardioembolic stroke	1,375 (98.00)	36,020 (96.70)		1,373 (98.00)	27,533 (98.49)	
Cardioembolic stroke	28 (2.00)	1,229 (3.30)		28 (2.00)	422 (1.51)	
Comorbidity						
Patients (missing cases)	1,403 (0)	37,249 (0)	<0.001^#^	1,401 (0)	27,955 (0)	<0.001^#^
No	695 (49.54)	15,210 (40.83)		694 (49.54)	11,746 (42.02)	
Yes	708 (50.46)	22,039 (59.17)		707 (50.46)	16,209 (57.98)	
Concomitant medication						
Patients (missing cases)	1,403 (0)	37,249 (0)	0.003^#^	1,401 (0)	27,955 (0)	0.380^#^
No	200 (14.26)	4,347 (11.67)		200 (14.28)	3,761 (13.45)	
Yes	1,203 (85.74)	32,902 (88.33)		1,201 (85.72)	24,194 (86.55)	

The percentage calculation was based on the non-missing data if there were any missing cases; otherwise, based on the total number of patient cases.

^*^ Comparison using t-test.

^#^ Comparison using χ2 test.

PSM, propensity score matching; SD, standard deviation; Q, quartile. Comorbidity included hypertension, hyperlipidemia, diabetes, coronary heart disease, and atrial fibrillation, et al. Concomitant medication included statins, anticoagulants, and proton pump inhibitors, et al.

### 3.3 MACCE after 1:20 PSM

The 1-year MACCE incidence was lower in the observational group than in the control group (14.99% vs. 18.71%) (*P* < 0.001). Additionally, the 2-year (19.41% vs. 23.85%) and 3-year (21.27% vs. 26.57%) MACCE incidence was also lower in the observational group than in the control group (both *P* < 0.001) (Table 2).

**TABLE 2:** Occurrence of MACCE.

Items	Observational group (N = 1,401)	Control group (N = 27,955)	*P* value
1-year MACCE			
Patients/total patients	210/1,401	5,230/27,955	<0.001
Percentage (95% CI)	14.99 (13.16, 16.97)	18.71 (18.25, 19.17)	
2-year MACCE			
Patients/total patients	272/1,401	6,667/27,955	<0.001
Percentage (95% CI)	19.41 (17.37, 21.58)	23.85 (23.35, 24.35)	
3-year MACCE			
Patients/total patients	298/1,401	7,427/27,955	<0.001
Percentage (95% CI)	21.27 (19.15, 23.51)	26.57 (26.05, 27.09)	

MACCE, major adverse cardiovascular and cerebrovascular event; CI, confidence interval.

**FIGURE 2:**
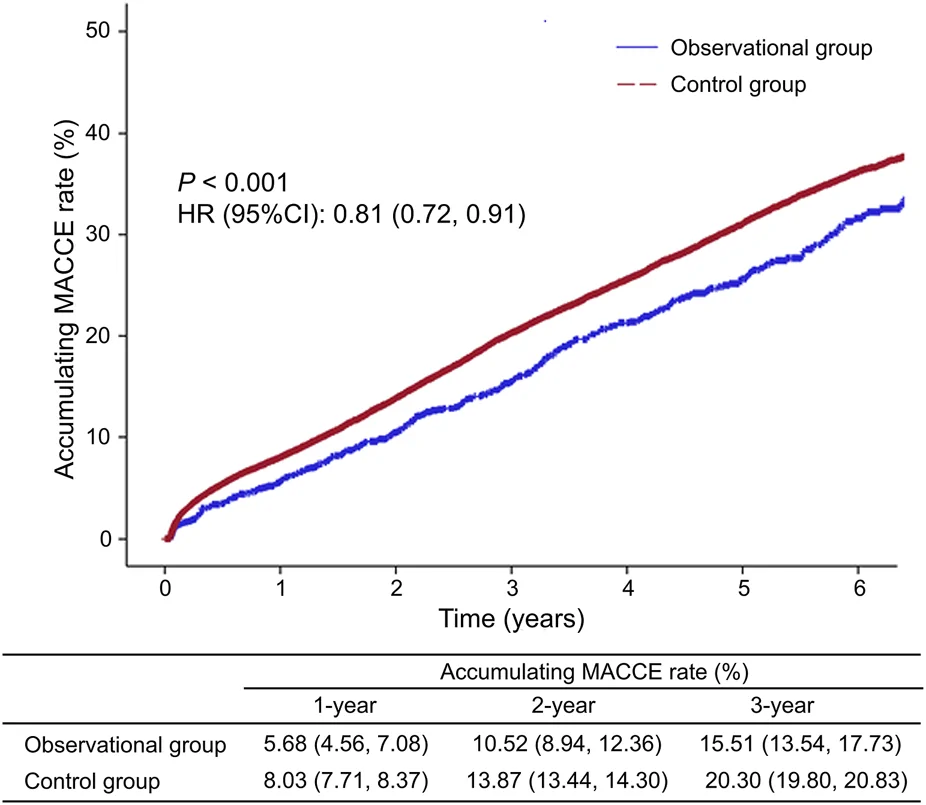
Accumulating MACCE in observation and control groups after 1:20 PSM.

The observational group (vs. the control group) was related to decreased accumulating MACCE [hazard ratio (HR): 0.81, *P* < 0.001]. The 1-, 2-, and 3-year accumulating MACCE rates were 5.68%, 10.52%, and 15.51% in the observational group, which were 8.03%, 13.87%, and 20.30% in the control group (Figure 2).

### 3.4 Mortality and visit to rehabilitation department or rehabilitation hospital after 1:20 PSM

The 1-year all-cause mortality incidence did not differ between the observational and control groups (0.21% vs. 0.25%) (*P* = 1.000). In addition, no difference was observed in the rate of 1-year visits to the rehabilitation department or rehabilitation hospital between the two groups (3.07% vs. 3.97%) (*P* = 0.090) (Table 3).

**TABLE 3:** Occurrence of mortality and visit to rehabilitation department or rehabilitation hospital.

Items	Observational group (N = 1,401)	Control group (N = 27,955)	*P* value
1-year all-cause mortality			
Patients/total patients	3/1,401	70/27,955	1.000
Percentage (95% CI)	0.21 (0.04, 0.62)	0.25 (0.20, 0.32)	
1-year visit to rehabilitation department or rehabilitation hospital			
Patients/assessed patients	43/1,401	1,110/27,955	0.090
Percentage (95% CI)	3.07 (2.23, 4.11)	3.97 (3.74, 4.21)	

CI, confidence interval.

### 3.5 Subgroup analyses for 1-year MACCE and all-cause mortality stratified by clinical features after 1:20 PSM

The 1-year MACCE incidence was lower in the observational group than in the control group in patients aged 45–64 years (*P* < 0.001, adjusted *P* = 0.006), but not in patients aged <45 years and ≥65 years (both *P* > 0.05). In patients with non-cardioembolic stroke (*P* = 0.002, adjusted *P* = 0.009) and cardioembolic stroke (*P* = 0.007, adjusted *P* = 0.018), the 1-year MACCE incidence was lower in the observational group than in the control group. Only in patients with the duration from stroke occurrence to treatment within 2 weeks, the 1-year MACCE incidence was lower in the observational group than in the control group (*P* = 0.007, adjusted *P* = 0.018); however, this trend was not found in patients with the duration from stroke occurrence to treatment within 2 weeks-1 month, 1 month-3 months, 3 months–6 months, and >6 months (all *P* > 0.05). The reduction of 1-year MACCE incidence in the observational group vs. control group was only discovered in patients with the duration from treatment to MACCE <1 month (*P* < 0.001, adjusted *P* = 0.006), but not in patients with the duration from treatment to MACCE within 1 month–2 months, 2 months–3 months, 3 months–6 months, and ≥6 months (all *P* > 0.05) (Supplementary Table S1).

Regarding the 1-year all-cause mortality, it was not different between the two groups, regardless of age, type of stroke, duration from stroke occurrence to treatment, and duration from treatment to MACCE (all *P* > 0.05) (Supplementary Table S1).

### 3.6 Subgroup analyses for 1-year MACCE and all-cause mortality stratified by comorbidities after 1:20 PSM

In patients without hypertension (*P* = 0.003, adjusted *P* = 0.012), hyperlipidemia (*P* < 0.001, adjusted *P* = 0.006), diabetes (P = 0.007, adjusted *P* = 0.018), or coronary heart disease (*P* < 0.001, adjusted *P* = 0.006), the 1-year MACCE incidence was lower in the observational group than in the control group. Of note, although a significant reduction in 1-year MACCE incidence was observed among patients with diabetes before adjustment (*P* = 0.033), this difference did not remain statistically significant after FDR correction (adjusted *P* = 0.076) (Supplementary Table S2).

Regarding the 1-year all-cause mortality, it did not differ between the two groups, regardless of hypertension, hyperlipidemia, diabetes, and coronary heart disease (all *P* > 0.05) (Supplementary Table S2).

### 3.7 Safety after 1:20 PSM

The incidence of AESI was lower in the observational group than in the control group (18.56% vs. 21.82%) (*P* = 0.004). Specifically, the incidence of nausea (3.71% vs. 5.38%) (*P* = 0.007), gastrointestinal bleeding (1.64% vs. 2.73%) (*P* = 0.014), and other gastrointestinal discomforts other than nausea (17.20% vs. 19.71) (*P* = 0.021) was lower in the observational group than in the control group. Among other gastrointestinal discomforts other than nausea, the incidence of vomiting was decreased in the observation group compared with the control group (4.21% vs. 6.05%) (*P* = 0.005) (Table 4).

**TABLE 4:** AESI.

Items	Observational group (N = 1,401)			Control group (N = 27,955)			*P* value
	Number of events	Number of patients	Percentage	Number of events	Number of patients	Percentage	
AESI	576	260	18.56	13,307	6,101	21.82	0.004
Nausea	61	52	3.71	1,739	1,503	5.38	0.007
Gastrointestinal bleeding	49	23	1.64	1,300	762	2.73	0.014
Other gastrointestinal discomforts other than nausea	466	241	17.20	10,268	5,510	19.71	0.021
Abdominal pain	249	155	11.06	5,178	3,135	11.21	0.861
Abdominal distension	98	64	4.57	1,922	1,186	4.24	0.556
Vomiting	70	59	4.21	2,013	1,692	6.05	0.005
Diarrhea	27	27	1.93	769	615	2.20	0.496
Loss of appetite	21	16	1.14	349	273	0.98	0.540
Others	1	1	0.07	37	37	0.13	1.000

AESI, adverse events of special interest.

### 3.8 Sensitivity analyses using 1:4 PSM

After 1:4 matching, no significant differences in age, sex, type of medical visit, comorbidity, and concomitant medication were found between the two groups (all *P* > 0.05). Of note, type of stroke differed between the two groups (*P* = 0.024) (Supplementary Table S3). Nevertheless, the love plot showed that the absolute standardized mean differences (SMDs) for all covariates were below the predefined threshold of 0.1, indicating adequate covariate balance between the two groups (Supplementary Figure S1A). Furthermore, the substantial overlap of the propensity score distributions after matching demonstrated good comparability between the matched groups and further supported the validity of the matching procedure (Supplementary Figure S1B).

Regarding the primary outcomes, the 1-year MACCE incidence remained lower in the observational group than in the control group after 1:4 matching (14.99% vs. 18.58%) (*P* = 0.002). A similar trend was observed for 2-year (19.41% vs. 24.07%) and 3-year (21.27% vs. 26.80%) MACCE incidence between the observational and control groups (both *P* < 0.001) (Supplementary Table S4). Additionally, the observational group was associated with reduced accumulating MACCE (HR: 0.81, *P* = 0.001) (Supplementary Figure S2). The 1-year all-cause mortality and the rate of 1-year visits to the rehabilitation department or rehabilitation hospital did not differ between the two groups (both *P* > 0.05) (Supplementary Table S5). To further address the statistically significant differences for some variables (comorbidities in the original 1:20 matched cohort; type of stroke in the 1:4 matched cohort), multivariable logistic regression analyses adjusting for baseline characteristics were performed. The observational group remained independently associated with a lower risk of 1-year MACCE in both the original 1:20 matched cohort and the revised 1:4 matched cohort (both *P* = 0.001), confirming the robustness of the association (Supplementary Table S6). Analysis of the individual components of 1-year MACCE showed that the observational group had lower incidences of 1-year myocardial infarction (0.86% vs. 1.80%), angina (1.28% vs. 3.08%), and cardiovascular/cerebrovascular stent surgery (0.71% vs. 2.32%) than the control group (all *P* < 0.05) (Supplementary Table S7).

## 4 Discussion

To the best of our knowledge, this was the first large-scale real-world study that explored the efficacy and safety of Xuesaitong soft capsule plus antiplatelet agents in ischemic stroke patients. Due to the retrospective design, it was impossible to achieve balance in baseline characteristics between groups. Therefore, we used PSM to control for confounding factors and balance baseline characteristics between groups. The main findings of this study were summarized as follows: (1) The 1-, 2-, and 3-year MACCE incidence was lower in the observational group than in the control group; however, 1-year all-cause mortality and visits to the rehabilitation department or rehabilitation hospital rates were not different between the two groups. (2) Subgroup analyses suggested that the better efficacy outcomes of the observational group over the control group were found in patients aged 45–64 years, with the duration from stroke occurrence to treatment within 2 weeks, with the duration from treatment to MACCE <1 month, without hypertension, without hyperlipidemia, and without coronary heart disease. (3) The incidence of AESI was lower in the observational group than in the control group. (4) After 1:4 matching, the efficacy outcomes remained largely consistent with those of the original 1:20 PSM analysis, with the observational group continuing to show lower MACCE incidence than the control group.

Atherosclerosis is the major reason for the occurrence of MACCE, primarily through the mechanisms of plaque rupture and subsequent atherothrombosis (Fan and Watanabe, 2022). Previous studies demonstrated that PNSs could attenuate atherosclerosis through several mechanisms, such as inhibiting inflammation, suppressing ubiquitin-specific peptidase 2, and decreasing vascular endothelial growth factor and nicotinamide adenine dinucleotide phosphate oxidase subunit four expression (Qiao et al., 2015; Yang et al., 2022; Zhao et al., 2024). Importantly, previous studies also revealed that the combination of PNSs and antiplatelet agents might have a synergistic effect on inhibiting the occurrence of MACCE (Cui et al., 2024; Sun et al., 2021; Wang et al., 2021). For instance, a previous study indicated that PNSs could cooperate with aspirin to treat blood stasis, which might further suppress the occurrence of MACCE (Sun et al., 2021). Additionally, PNSs plus aspirin inhibited cerebral ischemia-reperfusion injury in rats through the phosphoinositide 3-kinase/protein kinase C pathway, thereby potentially lowering MACCE (Cui et al., 2024). Moreover, PNSs plus aspirin potentiated the antiplatelet effect, which may further reduce the occurrence of MACCE (Wang et al., 2021). In the current study, we observed that Xuesaitong soft capsule plus antiplatelet agents reduced the occurrence of 1-, 2-, and 3-year MACCE compared to the antiplatelet agents alone in ischemic stroke patients. Additionally, this combined regimen was also related to lower accumulating MACCE occurrence. The better efficacy of Xuesaitong soft capsule plus antiplatelet agents might be attributed to their synergistic effects on enhancing the antiplatelet activity, ameliorating blood stasis, and mitigating cerebral ischemia-reperfusion injury (Cui et al., 2024; Sun et al., 2021; Wang et al., 2021). Overall, our findings suggested that Xuesaitong soft capsule plus antiplatelet agents could be recommended for ischemic stroke patients to prevent MACCE. Furthermore, we found that the 1-year all-cause mortality and visit to the rehabilitation department or rehabilitation hospital rates did not differ between the two groups. Further prospective studies or randomized controlled trials should be conducted to validate these findings.

Subgroup analyses based on clinical features and comorbidities were conducted to identify potential subpopulations that might benefit more from Xuesaitong soft capsule plus antiplatelet agents. Regarding clinical features, we found that in subgroups aged 45–64 years, with duration from stroke occurrence to treatment within 2 weeks, and with duration from treatment to MACCE <1 month, the 1-year MACCE incidence was lower in the observational group than in the control group. These findings suggested that the potential benefit of Xuesaitong soft capsule plus antiplatelet agents may vary according to patient characteristics and treatment timing. However, these subgroup findings should be considered exploratory and require confirmation in prospective studies. The presence of comorbidities may influence the efficacy of treatments in ischemic stroke (Cipolla et al., 2018; Paul and Candelario-Jalil, 2021). In this study, we discovered that only in subgroups without hypertension, hyperlipidemia, diabetes, or coronary heart disease, the 1-year MACCE incidence was lower in the observational group than in the control group. A potential reason might be that the presence of these comorbidities increased the vulnerability of the brain to ischemic damage, which might impair the efficacy of Xuesaitong soft capsule plus antiplatelet agents (Candelario-Jalil and Paul, 2021).

Despite the satisfactory effect of antiplatelet agents on inhibiting thrombogenesis, the use of these drugs carries the risk of gastrointestinal bleeding and discomfort (Greco et al., 2023; Willey and Chaturvedi, 2022). According to a previous study, dyspeptic symptoms were relieved in the PNS plus aspirin group compared to the aspirin group (Wang et al., 2021). In line with this previous study, we discovered that the incidence of nausea and other gastrointestinal discomforts, other than nausea (particularly vomiting), was lower in the observational group than in the control group. Additionally, the observational group exhibited a lower incidence of gastrointestinal bleeding than the control group. We speculated that Xuesaitong soft capsule plus antiplatelet agents might relieve gastrointestinal discomfort and bleeding through regulating the arachidonic acid/prostaglandin pathway or the thromboxane A2/prostaglandin I2 ratio (Wang et al., 2021; Xu et al., 2021). According to the product information, Xuesaitong soft capsule is generally well tolerated, with mild gastrointestinal symptoms (such as nausea and abdominal discomfort) being the commonly reported adverse reactions, while rare adverse events, such as skin reactions, dizziness, headache, and cardiovascular symptoms, have also been documented. Given its antiplatelet and blood-activating properties, potential interactions with anticoagulant or antiplatelet agents should be considered, particularly regarding an increased risk of bleeding. In the present study, no additional safety signals were observed with the combination of Xuesaitong soft capsule and antiplatelet agents, and the observational group even showed lower incidence of gastrointestinal bleeding compared with the control group. Overall, the combination of Xuesaitong soft capsule and antiplatelet agents was generally well tolerated and safe in ischemic stroke patients. However, appropriate clinical monitoring remains warranted during combined therapy.

To further examine the robustness of our findings, we conducted a sensitivity analysis using a 1:4 PSM strategy. The efficacy outcomes were highly consistent with those obtained from the original 1:20 matched cohort, with the observational group continuing to demonstrate lower 1-, 2-, and 3-year MACCE incidences than the control group. These findings indicated that the observed association between Xuesaitong soft capsule plus antiplatelet agents and a reduced risk of MACCE was robust and was not materially influenced by the matching strategy. Furthermore, analysis of the individual MACCE components showed that the observational group had lower incidences of 1-year myocardial infarction, angina, and cardiovascular/cerebrovascular stent surgery, whereas no significant differences were observed for the remaining individual endpoints. These results suggested that the overall reduction in MACCE was primarily driven by improvements in specific cardiovascular events rather than by uniform reductions across all MACCE components. It is noteworthy that although all covariates achieved absolute SMDs below 0.1 after 1:4 matching, a statistically significant difference in stroke type remained between the two groups. This finding might be attributable to the reduced size of the matched control group after changing the matching ratio from 1:20 to 1:4, while the matched cohort remained sufficiently large for statistical tests to detect very small absolute differences. Importantly, the absolute differences in the proportions of cardioembolic stroke (2.00% vs. 1.21%) and non-cardioembolic stroke (98.00% vs. 98.79%) were minimal, suggesting that this imbalance was unlikely to be clinically meaningful. To further address this statistically significant difference in stroke type, we additionally performed multivariable logistic regression analyses adjusting for baseline characteristics. The observational group remained independently associated with a lower risk of 1-year MACCE after adjustment, providing additional evidence that the observed association was robust.

Several limitations should be noted in this study. (1) This was a retrospective study. Therefore, selection bias might exist. Further randomized controlled trials are required to validate the findings of this study. (2) The 1-year all-cause mortality was relatively low in both groups. This may be related to the characteristics of the study population, as most patients were from outpatient settings and may have had relatively lower disease severity. Moreover, although mortality data were obtained from in-hospital death records and public health system records, incomplete ascertainment of mortality events cannot be completely excluded. The limited number of mortality events may have reduced the statistical power to detect between-group differences. Further studies with more comprehensive outcome ascertainment and longer follow-up are warranted to validate the impact of Xuesaitong soft capsule plus antiplatelet agents on all-cause mortality. (3) The rate of visits to the rehabilitation department or rehabilitation hospital might lack the ability to accurately reflect patients’ functional outcomes. Further studies should apply standardized measures, such as the modified Rankin Scale, to verify the effect of Xuesaitong soft capsule plus antiplatelet agents on functional outcomes in ischemic stroke patients.

## 5 Conclusion

Xuesaitong soft capsule combined with antiplatelet agents is associated with lower MACCE incidence and gastrointestinal bleeding and discomfort compared to antiplatelet agents alone in ischemic stroke patients. In clinical practice, Xuesaitong soft capsule could be used as an adjunctive therapy to antiplatelet agents for ischemic stroke patients.

## Data Availability

The original contributions presented in the study are included in the article/Supplementary Material, further inquiries can be directed to the corresponding authors.

## Supplementary material

Supplementary Figure S1 Covariate balance and propensity score distribution after 1:4 PSM. Love plot of absolute SMDs for baseline covariates before and after matching **(A)** Density plot of the propensity score distribution in the matched cohorts **(B)**.

Supplementary Figure S2 Accumulating MACCE in observation and control groups after 1:4 PSM.
