# Supplementary material for: Efficacy and safety of Xuesaitong soft capsule plus antiplatelet agents in ischemic stroke patients: a large-scale, multicenter, controlled, retrospective real-world study
Source: Front Pharmacol. 2026 Sep 3;17:1851121. doi: 10.3389/fphar.2026.1851121 (PMC13582177; doi:10.3389/fphar.2026.1851121)
Supplement: Supplementary file 1 [file DataSheet1.docx]

**Supplementary Table 1.** Subgroup analysis for 1-year MACCE and 1-year all-cause mortality based on age and disease-related characteristics.

| Items | 1-year MACCE | | | |  | 1-year all-cause mortality | | | |
| --- | --- | --- | --- | --- | --- | --- | --- | --- | --- |
|  | Observational group  (N = 1401) | Control group  (N = 27955) | *P* value | Adjusted *P* value |  | Observational group  (N = 1401) | Control group  (N = 27955) | *P* value | Adjusted *P* value |
| **Age** |  |  |  |  |  |  |  |  |  |
| <45 years |  |  | 0.229 | 0.310 |  |  |  | 1.000 | 1.000 |
| Patients/total patients | 7/74 | 244/1688 |  |  |  | 0/74 | 1/1688 |  |  |
| Percentage (95% CI) | 9.46 (3.89, 18.52) | 14.45 (12.81, 16.22) |  |  |  | 0.00 (0.00, 4.86) | 0.06 (0.00, 0.33) |  |  |
| 45-64 years |  |  | <0.001 | 0.006 |  |  |  | 0.200 | 1.000 |
| Patients/total patients | 82/712 | 2341/13886 |  |  |  | 2/712 | 15/13886 |  |  |
| Percentage (95% CI) | 11.52 (9.27, 14.09) | 16.86 (16.24, 17.49) |  |  |  | 0.28 (0.03, 1.01) | 0.11 (0.06, 0.18) |  |  |
| ≥65 years |  |  | 0.318 | 0.406 |  |  |  | 0.521 | 1.000 |
| Patients/total patients | 121/615 | 2645/12381 |  |  |  | 1/615 | 54/12381 |  |  |
| Percentage (95% CI) | 19.67 (16.60, 23.04) | 21.36 (20.64, 22.10) |  |  |  | 0.16 (0.00, 0.90) | 0.44 (0.33, 0.57) |  |  |
| **Type of stroke** |  |  |  |  |  |  |  |  |  |
| Non-cardioembolic stroke |  |  | 0.002 | 0.009 |  |  |  | 0.775 | 1.000 |
| Patients/total patients | 207/1373 | 5079/27533 |  |  |  | 2/1373 | 68/27533 |  |  |
| Percentage (95% CI) | 15.08 (13.22, 17.08) | 18.45 (17.99, 18.91) |  |  |  | 0.15 (0.02, 0.53) | 0.25 (0.19, 0.31) |  |  |
| Cardioembolic stroke |  |  | 0.007 | 0.018 |  |  |  | 0.176 | 1.000 |
| Patients/total patients | 3/28 | 151/422 |  |  |  | 1/28 | 2/422 |  |  |
| Percentage (95% CI) | 10.71 (2.27, 28.23) | 35.78 (31.20, 40.56) |  |  |  | 3.57 (0.09, 18.35) | 0.47 (0.06, 1.70) |  |  |
| **Duration from stroke occurrence to treatment** | | | |  |  |  |  |  |  |
| 2 weeks |  |  | 0.007 | 0.018 |  |  |  | 1.000 | 1.000 |
| Patients/total patients | 196/1320 | 4322/24326 |  |  |  | 3/1320 | 58/24326 |  |  |
| Percentage (95% CI) | 14.85 (12.97, 16.88) | 17.77 (17.29, 18.25) |  |  |  | 0.23 (0.05, 0.66) | 0.24 (0.18, 0.31) |  |  |
| 2 weeks-1 month |  |  | 0.164 | 0.269 |  |  |  | 1.000 | 1.000 |
| Patients/total patients | 3/26 | 222/959 |  |  |  | 0/26 | 5/959 |  |  |
| Percentage (95% CI) | 11.54 (2.45, 30.15) | 23.15 (20.51, 25.95) |  |  |  | 0.00 (0.00, 13.23) | 0.52 (0.17, 1.21) |  |  |
| 1 month-3 months |  |  | 0.385 | 0.466 |  |  |  | 1.000 | 1.000 |
| Patients/total patients | 6/34 | 344/1429 |  |  |  | 0/34 | 5/1429 |  |  |
| Percentage (95% CI) | 17.65 (6.76, 34.53) | 24.07 (21.88, 26.38) |  |  |  | 0.00 (0.00, 10.28) | 0.35 (0.11, 0.81) |  |  |
| 3 months-6 months |  |  | 1.000 | 1.000 |  |  |  | 1.000 | 1.000 |
| Patients/total patients | 4/17 | 245/946 |  |  |  | 0/17 | 2/946 |  |  |
| Percentage (95% CI) | 23.53 (6.81, 49.90) | 25.90 (23.13, 28.82) |  |  |  | 0.00 (0.00, 19.51) | 0.21 (0.03, 0.76) |  |  |
| >6 months |  |  | 1.000 | 1.000 |  |  |  | 1.000 | 1.000 |
| Patients/total patients | 1/4 | 97/295 |  |  |  | 0/4 | 0/295 |  |  |
| Percentage (95% CI) | 25.00 (0.63, 80.59) | 32.88 (27.55, 38.56) |  |  |  | 0.00 (0.00, 60.24) | 0.00 (0.00, 1.24) |  |  |
| **Duration from treatment to MACCE** | | |  |  |  |  |  |  |  |
| <1 month |  |  | <0.001 | 0.006 |  |  |  | 1.000 | 1.000 |
| Patients/total patients | 110/256 | 2920/2920 |  |  |  | 1/256 | 13/2920 |  |  |
| Percentage (95% CI) | 42.97 (36.82, 49.28) | 100.00 (99.87, 100.00) |  |  |  | 0.39 (0.01, 2.16) | 0.45 (0.24, 0.76) |  |  |
| 1 month-2 months |  |  | 0.207 | 0.310 |  |  |  | 1.000 | 1.000 |
| Patients/total patients | 59/585 | 1373/11629 |  |  |  | 1/585 | 34/11629 |  |  |
| Percentage (95% CI) | 10.09 (7.77, 12.82) | 11.81 (11.23, 12.41) |  |  |  | 0.17 (0.00, 0.95) | 0.29 (0.20, 0.41) |  |  |
| 2 months-3 months |  |  | 0.095 | 0.182 |  |  |  | 1.000 | 1.000 |
| Patients/total patients | 16/277 | 452/5230 |  |  |  | 0/277 | 10/5230 |  |  |
| Percentage (95% CI) | 5.78 (3.34, 9.21) | 8.64 (7.89, 9.44) |  |  |  | 0.00 (0.00, 1.32) | 0.19 (0.09, 0.35) |  |  |
| 3 months-6 months |  |  | 0.227 | 0.310 |  |  |  | 0.435 | 1.000 |
| Patients/total patients | 21/223 | 389/5357 |  |  |  | 1/223 | 13/5357 |  |  |
| Percentage (95% CI) | 9.42 (5.92, 14.03) | 7.26 (6.58, 7.99) |  |  |  | 0.45 (0.01, 2.47) | 0.24 (0.13, 0.41) |  |  |
| ≥6 months |  |  | 0.153 | 0.269 |  |  |  | 1.000 | 1.000 |
| Patients/total patients | 4/60 | 96/2819 |  |  |  | 0/60 | 0/2819 |  |  |
| Percentage (95% CI) | 6.67 (1.85, 16.20) | 3.41 (2.77, 4.14) |  |  |  | 0.00 (0.00, 5.96) | 0.00 (0.00, 0.13) |  |  |

MACCE, major adverse cardiovascular and cerebrovascular event; CI, confidence interval.

Adjusted *P* value was corrected using the BH-FDR (Benjamini-Hochberg False Discovery Rate) method.
