# Supplementary material for: Efficacy and safety of Xuesaitong soft capsule plus antiplatelet agents in ischemic stroke patients: a large-scale, multicenter, controlled, retrospective real-world study
Source: Front Pharmacol. 2026 Sep 3;17:1851121. doi: 10.3389/fphar.2026.1851121 (PMC13582177; doi:10.3389/fphar.2026.1851121)
Supplement: Supplementary file 2 [file DataSheet2.docx]

**Supplementary Table 2.** Subgroup analysis for 1-year MACCE and 1-year all-cause mortality based on comorbidities.

| Items | 1-year MACCE | | | |  | 1-year all-cause mortality | | | |
| --- | --- | --- | --- | --- | --- | --- | --- | --- | --- |
|  | Observational group  (N = 1401) | Control group  (N = 27955) | *P* value | Adjusted *P* value |  | Observational group  (N = 1401) | Control group  (N = 27955) | *P* value | Adjusted *P* value |
| **Hypertension** |  |  |  |  |  |  |  |  |  |
| With |  |  | 0.074 | 0.155 |  |  |  | 0.705 | 1.000 |
| Patients/total patients | 104/619 | 2687/13631 |  |  |  | 2/619 | 40/13631 |  |  |
| Percentage (95% CI) | 16.80 (13.94, 19.98) | 19.71 (19.05, 20.39) |  |  |  | 0.32 (0.04, 1.16) | 0.29 (0.21, 0.40) |  |  |
| Without |  |  | 0.003 | 0.012 |  |  |  | 1.000 | 1.000 |
| Patients/total patients | 106/782 | 2543/14324 |  |  |  | 1/782 | 30/14324 |  |  |
| Percentage (95% CI) | 13.55 (11.23, 16.16) | 17.75 (17.13, 18.39) |  |  |  | 0.13 (0.00, 0.71) | 0.21 (0.14, 0.30) |  |  |
| **Hyperlipidemia** |  |  |  |  |  |  |  |  |  |
| With |  |  | 0.789 | 0.907 |  |  |  | 1.000 | 1.000 |
| Patients/total patients | 4/36 | 101/944 |  |  |  | 0/36 | 1/944 |  |  |
| Percentage (95% CI) | 11.11 (3.11, 26.06) | 10.70 (8.80, 12.85) |  |  |  | 0.00 (0.00, 9.74) | 0.11 (0.00, 0.59) |  |  |
| Without |  |  | <0.001 | 0.006 |  |  |  | 1.000 | 1.000 |
| Patients/total patients | 206/1365 | 5129/27011 |  |  |  | 3/1365 | 69/27011 |  |  |
| Percentage (95% CI) | 15.09 (13.23, 17.10) | 18.99 (18.52, 19.46) |  |  |  | 0.22 (0.05, 0.64) | 0.26 (0.20, 0.32) |  |  |
| **Diabetes** |  |  |  |  |  |  |  | 1.000 | 1.000 |
| With |  |  | 0.033 | 0.076 |  |  |  |  |  |
| Patients/total patients | 28/159 | 1027/4101 |  |  |  | 0/159 | 12/4101 |  |  |
| Percentage (95% CI) | 17.61 (12.03, 24.44) | 25.04 (23.72, 26.40) |  |  |  | 0.00 (0.00, 2.29) | 0.29 (0.15, 0.51) |  |  |
| Without |  |  | 0.007 | 0.018 |  |  |  | 1.000 | 1.000 |
| Patients/total patients | 182/1242 | 4203/23854 |  |  |  | 3/1242 | 58/23854 |  |  |
| Percentage (95% CI) | 14.65 (12.73, 16.74) | 17.62 (17.14, 18.11) |  |  |  | 0.24 (0.05, 0.70) | 0.24 (0.18, 0.31) |  |  |
| **Coronary heart disease** |  |  |  |  |  |  |  |  |  |
| With |  |  | 0.951 | 1.000 |  |  |  | 1.000 | 1.000 |
| Patients/total patients | 30/158 | 762/4055 |  |  |  | 0/158 | 14/4055 |  |  |
| Percentage (95% CI) | 18.99 (13.19, 25.98) | 18.79 (17.60, 20.03) |  |  |  | 0.00 (0.00, 2.31) | 0.35 (0.19, 0.58) |  |  |
| Without |  |  | <0.001 | 0.006 |  |  |  | 0.767 | 1.000 |
| Patients/total patients | 180/1243 | 4468/23900 |  |  |  | 3/1243 | 56/23900 |  |  |
| Percentage (95% CI) | 14.48 (12.57, 16.56) | 18.69 (18.20, 19.19) |  |  |  | 0.24 (0.05, 0.70) | 0.23 (0.18, 0.30) |  |  |

MACCE, major adverse cardiovascular and cerebrovascular event; CI, confidence interval.

Adjusted *P* value was corrected using the BH-FDR (Benjamini-Hochberg False Discovery Rate) method.
