# Supplementary material for: Efficacy and safety of Xuesaitong soft capsule plus antiplatelet agents in ischemic stroke patients: a large-scale, multicenter, controlled, retrospective real-world study
Source: Front Pharmacol. 2026 Sep 3;17:1851121. doi: 10.3389/fphar.2026.1851121 (PMC13582177; doi:10.3389/fphar.2026.1851121)
Supplement: Supplementary file 3 [file DataSheet3.docx]

**Supplementary Table 3.** Clinical characteristics after PSM with a 1:4 ratio.

| Items | Observational group  (N = 1401) | Control group  (N = 5604) | *P* value |
| --- | --- | --- | --- |
| Age (years) |  |  | 0.716^*^ |
| Patients (missing cases) | 1401 (0) | 5604 (0) |  |
| Mean (SD) | 62.6 (11.3) | 62.7 (11.3) |  |
| Median (Q1, Q3) | 63.0 (55.0, 70.0) | 63.0 (55.0, 70.0) |  |
| Min, max | 31, 120 | 27, 120 |  |
| Sex |  |  | 0.821^#^ |
| Patients (missing cases) | 1401 (0) | 5604 (0) |  |
| Male | 916 (65.38) | 3646 (65.06) |  |
| Female | 485 (34.62) | 1958 (34.94) |  |
| Type of medical visit |  |  | 0.995^#^ |
| Patients (missing cases) | 1401 (0) | 5604 (0) |  |
| Emergency‌ | 291 (20.77) | 1159 (20.68) |  |
| Outpatient‌ | 803 (57.32) | 3220 (57.46) |  |
| Inpatient‌ | 307 (21.91) | 1225 (21.86) |  |
| Type of stroke |  |  | 0.024^#^ |
| Patients (missing cases) | 1401 (0) | 5604 (0) |  |
| Non-cardioembolic stroke | 1373 (98.00) | 5536 (98.79) |  |
| Cardioembolic stroke | 28 (2.00) | 68 (1.21) |  |
| Comorbidity |  |  | 0.802^#^ |
| Patients (missing cases) | 1401 (0) | 5604 (0) |  |
| No | 694 (49.54) | 2755 (49.16) |  |
| Yes | 707 (50.46) | 2849 (50.84) |  |
| Concomitant medication |  |  | 1.000^#^ |
| Patients (missing cases) | 1401 (0) | 5604 (0) |  |
| No | 200 (14.28) | 800 (14.28) |  |
| Yes | 1201 (85.72) | 4804 (85.72) |  |

The percentage calculation was based on the non-missing data if there were any missing cases; otherwise, based on the total number of patient cases.

^*^, comparison using t-test; ^#^, comparison using χ^2^ test. PSM, propensity score matching; SD, standard deviation; Q, quartile.

Comorbidity included hypertension, hyperlipidemia, diabetes, coronary heart disease, and atrial fibrillation, et al.

Concomitant medication included statins, anticoagulants, and proton pump inhibitors, et al.
