# Supplementary material for: Efficacy and safety of Xuesaitong soft capsule plus antiplatelet agents in ischemic stroke patients: a large-scale, multicenter, controlled, retrospective real-world study
Source: Front Pharmacol. 2026 Sep 3;17:1851121. doi: 10.3389/fphar.2026.1851121 (PMC13582177; doi:10.3389/fphar.2026.1851121)
Supplement: Supplementary file 4 [file DataSheet4.docx]

**Supplementary Table 4.** Occurrence of MACCE after PSM with a 1:4 ratio.

| Items | Observational group  (N = 1401) | Control group  (N = 5604) | *P* value |
| --- | --- | --- | --- |
| 1-year MACCE |  |  | 0.002^#^ |
| Patients/total patients | 210/1401 | 1041/5604 |  |
| Percentage (95% CI) | 14.99 (13.16, 16.97) | 18.58 (17.57, 19.62) |  |
| 2-year MACCE |  |  | <0.001^#^ |
| Patients/total patients | 272/1401 | 1349/5604 |  |
| Percentage (95% CI) | 19.41 (17.37, 21.58) | 24.07 (22.96, 25.21) |  |
| 3-year MACCE |  |  | <0.001^#^ |
| Patients/total patients | 298/1401 | 1502/5604 |  |
| Percentage (95% CI) | 21.27 (19.15, 23.51) | 26.80 (25.65, 27.98) |  |

MACCE, major adverse cardiovascular and cerebrovascular event; PSM, propensity score matching; CI, confidence interval.

^#^, comparison using χ^2^ test.
