# Supplementary material for: Efficacy and safety of Xuesaitong soft capsule plus antiplatelet agents in ischemic stroke patients: a large-scale, multicenter, controlled, retrospective real-world study
Source: Front Pharmacol. 2026 Sep 3;17:1851121. doi: 10.3389/fphar.2026.1851121 (PMC13582177; doi:10.3389/fphar.2026.1851121)
Supplement: Supplementary file 5 [file DataSheet5.docx]

**Supplementary Table 5.** Occurrence of mortality and visit to rehabilitation department or rehabilitation hospital after PSM with a 1:4 ratio.

| Items | Observational group  (N = 1401) | Control group  (N = 5604) | *P* value |
| --- | --- | --- | --- |
| 1-year all-cause mortality |  |  | 0.452^&^ |
| Patients/total patients | 3/1401 | 21/5604 |  |
| Percentage (95% CI) | 0.21 (0.04, 0.62) | 0.37 (0.23, 0.57) |  |
| 1-year visit to rehabilitation department or rehabilitation hospital | | | 0.202^#^ |
| Patients/assessed patients | 43/1401 | 212/5604 |  |
| Percentage (95% CI) | 3.07 (2.23, 4.11) | 3.78 (3.30, 4.32) |  |

PSM, propensity score matching; CI, confidence interval.

^#^, comparison using χ^2^ test, ^&^, comparison using Fisher's exact test.
