# Supplementary material for: Efficacy and safety of Xuesaitong soft capsule plus antiplatelet agents in ischemic stroke patients: a large-scale, multicenter, controlled, retrospective real-world study
Source: Front Pharmacol. 2026 Sep 3;17:1851121. doi: 10.3389/fphar.2026.1851121 (PMC13582177; doi:10.3389/fphar.2026.1851121)
Supplement: Supplementary file 6 [file DataSheet6.docx]

**Supplementary Table 6.** Multivariate logistic regression for 1-year MACCE.

| Group (Observational group vs. Control group) | OR (95% CI) | *P* value |
| --- | --- | --- |
| PSM 1:20 (N = 29356) | 0.763 (0.655, 0.890) | 0.001 |
| PSM 1:4 (N = 7005) | 0.761 (0.645, 0.898) | 0.001 |

MACCE, major adverse cardiovascular and cerebrovascular event; PSM, propensity score matching; CI, confidence interval.

Age, gender, type of visit, type of stroke, comorbidity, and concomitant medication were adjusted in models.
