# Supplementary material for: Efficacy and safety of Xuesaitong soft capsule plus antiplatelet agents in ischemic stroke patients: a large-scale, multicenter, controlled, retrospective real-world study
Source: Front Pharmacol. 2026 Sep 3;17:1851121. doi: 10.3389/fphar.2026.1851121 (PMC13582177; doi:10.3389/fphar.2026.1851121)
Supplement: Supplementary file 7 [file DataSheet7.docx]

**Supplementary Table 7.** Individual components of 1-year MACCE after PSM with a 1:4 ratio.

| Items | Observational group (N = 1401) | Control group (N = 5604) | *P* value |
| --- | --- | --- | --- |
| 1-year MACCE |  |  | 0.002^#^ |
| Patients/total patients | 210/1401 | 1041/5604 |  |
| Percentage (95% CI) | 14.99 (13.16, 16.97) | 18.58 (17.57, 19.62) |  |
| 1-year ischemic stroke |  |  | 0.105^#^ |
| Patients/total patients | 187/1401 | 844/5604 |  |
| Percentage (95% CI) | 13.35 (11.61, 15.24) | 15.06 (14.13, 16.02) |  |
| 1-year hemorrhagic stroke |  |  | 0.241^#^ |
| Patients/total patients | 14/1401 | 39/5604 |  |
| Percentage (95% CI) | 1.00 (0.55, 1.67) | 0.70 (0.50, 0.95) |  |
| 1-year transient ischemic attack |  |  | 0.933^#^ |
| Patients/total patients | 7/1401 | 29/5604 |  |
| Percentage (95% CI) | 0.50 (0.20, 1.03) | 0.52 (0.35, 0.74) |  |
| 1-year myocardial infarction |  |  | 0.012^#^ |
| Patients/total patients | 12/1401 | 101/5604 |  |
| Percentage (95% CI) | 0.86 (0.44, 1.49) | 1.80 (1.47, 2.19) |  |
| 1-year angina |  |  | <0.001^#^ |
| Patients/total patients | 18/1401 | 170/5604 |  |
| Percentage (95% CI) | 1.28 (0.76, 2.02) | 3.03 (2.60, 3.52) |  |
| 1-year acute coronary syndrome |  |  | 1.000^#^ |
| Patients/total patients | 7/1401 | 28/5604 |  |
| Percentage (95% CI) | 0.50 (0.20, 1.03) | 0.50 (0.33, 0.72) |  |
| 1-year use of thrombolytic drugs |  |  | (-) |
| Patients/total patients | 0/1401 | 0/5604 |  |
| Percentage (95% CI) | 0.00 (0.00, 0.26) | 0.00 (0.00, 0.07) |  |
| 1-year cardiovascular/cerebrovascular stent surgery |  |  | <0.001^#^ |
| Patients/total patients | 10/1401 | 130/5604 |  |
| Percentage (95% CI) | 0.71 (0.34, 1.31) | 2.32 (1.94, 2.75) |  |
| 1-year vascular recanalization therapy |  |  | 0.397^#^ |
| Patients/total patients | 5/1401 | 30/5604 |  |
| Percentage (95% CI) | 0.36 (0.12, 0.83) | 0.54 (0.36, 0.76) |  |

MACCE, major adverse cardiovascular and cerebrovascular event; PSM, propensity score matching; CI, confidence interval.

^#^, comparison using χ^2^ test.
